# Identification of Genes Coding Aminoglycoside Modifying Enzymes in* E. coli* of UTI Patients in India

**DOI:** 10.1155/2016/1875865

**Published:** 2016-06-15

**Authors:** Abdul Rouf Mir, Yasir Bashir, Firdous Ahmad Dar, M. Sekhar

**Affiliations:** ^1^Department of Biotechnology, Government Degree College Baramulla, Kashmir 193101, India; ^2^Department of Molecular Biology and Biotechnology, Tezpur University, Assam, India; ^3^Department of Biotechnology, The New College, Chennai, India

## Abstract

This study is to probe the pattern of antibiotic resistance against aminoglycosides and its mechanism in* E. coli* obtained from patients from Chennai, India. Isolation and identification of pathogens were done on MacConkey agar. Antimicrobial sensitivity testing was done by disc diffusion test. The identification of genes encoding aminoglycoside modifying enzymes was done by Polymerase Chain Reaction (PCR). Out of 98 isolates, 71 (72.45%) isolates were identified as* E. coli* and the remaining 27 (27.55%) as other bacteria. Disc diffusion method results showed a resistance level of 72.15% for streptomycin, 73.4% for gentamicin, 63.26% for neomycin, 57.14% for tobramycin, 47.9% for netilmicin, and 8.16% for amikacin in* E. coli*. PCR screening showed the presence of four genes, namely,* rrs*,* aacC2*,* aacA-aphD*, and* aphA3*, in their plasmid DNA. The results point towards the novel mechanism of drug resistance in* E. coli* from UTI patients in India as they confirm the presence of genes encoding enzymes that cause resistance to aminoglycoside drugs. This could be an alarm for drug prescription to UTI patients.

## 1. Introduction

Infectious diseases continue to be a leading cause of mortality all over the world especially in developing countries with poorly accessed health services [1]. With the passage of time, the burden of bacterial infections is showing an ascending trend and this is largely due to the generation of resistance mechanisms by bacteria [2, 3]. Among the various patterns of resistance, reports across the globe continue to emerge on the resistance to aminoglycoside antibiotics which otherwise have been reported as highly potent drugs against life threatening Gram-negative bacterial infections [4, 5]. Aminoglycosides act primarily by impairing bacterial protein synthesis through binding to prokaryotic ribosomes via 16S ribosomal RNA (16S rRNA) and by disrupting the bacterial cell membrane integrity [6]. Aminoglycoside resistance has been reported in both Gram-negative and Gram-positive bacteria and main mechanisms that affect the efficacy of aminoglycoside drugs are a decreased uptake and/or accumulation of the drug in bacteria and the expression of aminoglycoside modifying enzymes (AMEs) that eventually inactivate the drugs [7]. Reduced drug uptake and active efflux of aminoglycosides have emerged as an additional mechanism of aminoglycoside resistance in Gram-negative bacteria [6]. Enzymatic inactivation of aminoglycoside drugs is caused by acetyltransferases, nucleotidyltransferases, and phosphotransferases through acetylation, adenylation, and phosphorylation, respectively. The genetic determinants of these enzymes are often located on mobile elements facilitating the rapid dissemination of the genes in various bacterial populations [8]. The emergence of complexity in defence mechanism has intensified the epidemiological research on these antibiotics and various reports have emerged to document the resistance patterns and the reasoning behind that. The comparative studies on the data on mechanisms of aminoglycoside resistance in bacteria isolated from various regions of the world have helped in understanding the spread of multidrug-resistant strains [9]. Similar studies have correlated the selective pressure of antibiotics and the patterns of combinations of aminoglycoside resistance mechanisms [10]. Resistance patterns and prevalence of the aminoglycoside modifying enzymes in clinical isolates showing multidrug-resistant patterns have also been reported from various parts of India and it has been shown that high level aminoglycoside resistance genes are widely disseminated among Indian populations [11, 12]. Though aminoglycoside drugs are being widely prescribed to patients in India, there is urgent need for assessment of emerging drug resistance pattern against these drugs. This study aims at studying antibiotic resistance pattern on commonly used aminoglycoside antibiotics in India using disc diffusion and molecular screening method (PCR) to detect the aminoglycoside resistance genes* rrs* (*rrs1* and* rrs2*),* aacC2* (*aacC1-1*,* aacC1-2*,* aacC2-1*,* aacC2-2*,* aacC3-1*,* aacC3-2*,* aacC4-1*,* aacC4-2*,* aadC-1*, and* aadC-2*),* aacA-aphD* (*aacA-aphD-1* and* aacA-aphD-2*), and* aphA3* (*aphA3-1* and* aphA3-2*) in* E. coli* using eight sets of primers (Table 1). The same genes were earlier reported in bacteria of clinical isolates from Jordan University Hospital [13].

## 2. Materials and Methods

The study includes nonhospitalized patients attending clinical laboratory (Ehrlich Laboratory, Chennai, India). The clinical specimens were the urine samples collected from different patients from February to June 2015. The clean catch midstream urine samples were collected in sterile containers and transported within half an hour of collection to the laboratory. This study was duly approved by the Institutional Ethical Committee at University of Madras. Urine samples were collected from the patients after informed verbal consent. The mode of consent was duly approved by the ethical committee. A proper record of all the patients and healthy individuals has been maintained.

### 2.1. Isolation and Identification of Bacteria

Primary isolation was done on blood agar and MacConkey agar. Colonies from the primary isolation plates were picked up and Gram staining was done to study the morphology and Gram character.* E. coli* screening was carried out by carbohydrate fermentation test and the isolates were confirmed for genus and species by standard protocols [14]. Microscopic analysis was also used in identification.

### 2.2. Antibiotic Sensitivity Testing (Disc Diffusion Method)

Antimicrobial sensitivity testing was done by Kirby-Bauer Antimicrobial Susceptibility Test (disc diffusion method) using Mueller-Hinton agar [15]. Six different antibiotics were tested and the zone size was measured. Amikacin, streptomycin, gentamicin, tobramycin, netilmicin, and neomycin (obtained from HiMedia, India) were taken and screening test for detection of high level aminoglycoside resistance (HLAR) in* Enterococcus *species was confirmed as per Clinical and Laboratory Standards Institute (CLSI) approved standards [16] The* E. coli* isolates were described as isolates with high level aminoglycoside resistance considering growth ≥2048 *μ*g/mL for streptomycin, ≥512 *μ*g/mL for gentamicin, ≥512 *μ*g/mL for neomycin, ≥256 *μ*g/mL for tobramycin, ≥256 *μ*g/mL for netilmicin, and ≥256 *μ*g/mL for amikacin. MICs were determined by Etest (AB Biodisk).

### 2.3. Isolation of Plasmid DNA

Procedure for the isolation of plasmid DNA from Gram-negative bacteria that showed resistance to at least two aminoglycoside antibiotics (38 samples) was followed as given in HiElute Miniprep DNA Isolation Kit from HiMedia, India. The isolated plasmids were stored at −20°C in deep freezer till processed.

### 2.4. Identification of Genes Encoding Aminoglycoside Enzymes

Detection of aminoglycoside resistance genes* rrs *(*rrs1 *and* rrs2*)*, aacC2 *(*aacC1-1*,* aacC1-2*,* aacC2-1*,* aacC2-2*,* aacC3-1*,* aacC3-2*,* aacC4-1*,* aacC4-2*,* aadC-1*, and* aadC-2*),* aacA-aphD *(*aacA-aphD-1* and* aacA-aphD-2*), and* aphA3 *(*aphA3-1 *and* aphA3-2*) from* E. coli* was done by PCR technique using Thermal Cycler (PTC 150, MJ Research). Blue mix DNA polymerase master mix, deoxynucleotide triphosphates (dNTPs),* taq* DNA polymerase, and the reaction buffer containing magnesium ions and the other required components were obtained from RBC Bioscience, India. Primers for the genes were purchased from Invitrogen (USA) through JOYVEL Biotech, India. Template DNA was isolated from* E. coli*. The working concentration of the primer was taken as 200 mM. The amount of PCR reaction mixture was taken as 50 *μ*L which included 25 *μ*L of blue mix DNA master mix, 5 *μ*L of forward primer, 5 *μ*L of reverse primer, 5 *μ*L of template DNA, and 10 *μ*L of TAE buffer. The PCR was performed with initial denaturation at 95°C for 3 min followed by 32 cycles each of denaturation at 94°C for 30 sec, annealing at 60°C for 45 sec, and extension at 72°C for two min for each gene.

The amplification products were analyzed by 0.8% agarose gel electrophoresis and the product size was compared with DNA markers. After treatment with ethidium bromide (0.5 *μ*g/mL), the gels were visualized using gel-doc system (Bio-Rad Laboratories, USA).

## 3. Results

Out of the total 320 clinical specimens collected from a private clinical laboratory, Ehrlich Laboratory, only 98 (30.6%) showed pathogenic growth. Out of 98 isolates, 71 (72.45%) isolates were identified as* E. coli* and the remaining 27 (27.55%) as other bacteria.

Phenotypic resistance to aminoglycoside antibiotics as analyzed by standard Kirby-Bauer disc diffusion method showed an overall mean resistance level of 73.4% for gentamicin, 72.15% for streptomycin, 63.26% for neomycin, 57.14% for tobramycin, 47.9% for netilmicin, and 8.16% for amikacin.

**Figure 1:**
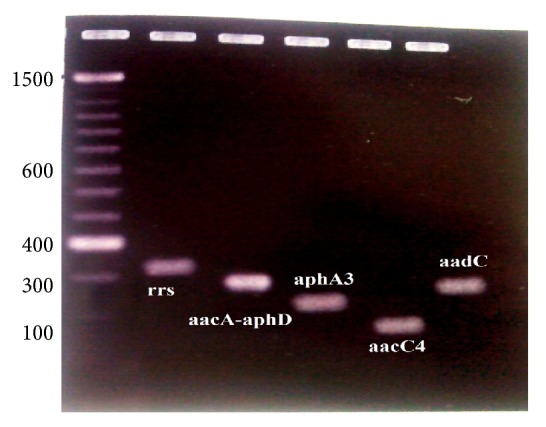
Gel documentation for genes encoding aminoglycoside modifying enzymes.

**Figure 2:**
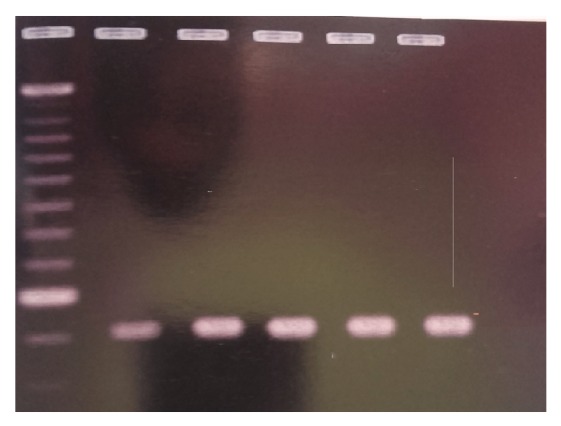
Gel documentation for* rrs* genes (five different samples) encoding aminoglycoside modifying enzymes.

We further probed the mechanism of resistance at molecular level using PCR technique for those* E. coli* isolates that were resistant to at least two aminoglycoside antibiotics. These included 38 of the total* E. coli* samples. PCR was carried out using eight sets of primers from previously published data (Table 1) [13]. The screened isolates were shown to carry four genes, namely,* rrs*,* aacC2*,* aacA-aphD*,and* aphA3*. Primers for five subtypes of the same gene* aacC* were employed. The* rrs* was found to be the most abundant and was found in 26 (68.4%) isolates and others included* aacC* gene in 18 (47.36%),* aphA3* gene in 13 (34.2%), and* aacC-aphD *gene in 7 (18.42%). No amplification of genes could be seen for seven isolates (Figures 1 and 2). We could find five isolates with three genes encoding aminoglycoside enzymes, sixteen isolates containing two genes, five isolates containing no genes, and other isolates containing a single gene. None of the isolates contained all the four genes in the plasmid genome. The 38 samples that were rendered to PCR analysis included patients aged 3–18 years (3 males and 5 females), 25–45 years (4 males and 12 females), and 55–80 years (5 males and 9 females). The presence of three genes in* E. coli* isolates was found in single male (age: 45 years) and four females (age: 8, 36, 68, and 72 years).

## 4. Discussion

Aminoglycoside resistance rates, phenotypes, and mechanisms of Gram-negative bacteria from infected patients across the world have shown progressive trends and it has been observed that resistance patterns have been disseminated in various bacterial species [7, 11]. Since aminoglycoside drugs are thus far making a choice for clinicians for infectious treatments, it is highly significant to investigate the emerging resistance patterns in bacteria against these drugs, so as to understand the current resistance status and explore the more efficient means of dealing with these infectious agents. We screened resistance to aminoglycoside antibiotics using both phenotypic and genotypic methods. These results clearly indicate the enhanced resistance in the bacterial isolates against various aminoglycoside antibiotics. An earlier report that has employed Kirby-Bauer method to check the high level aminoglycoside resistance in* Enterococci* isolates has provided the similar inference for aminoglycoside resistance against gentamicin, streptomycin, and both of them together from Chennai, India [11]. Another report from infected patients in Upper Egypt has shown Gram-negative bacterial resistance against streptomycin, neomycin, kanamycin, gentamicin, and tobramycin. Similar to our observation, minimal resistance was reported against amikacin antibiotic [7]. Other various similar reports have published data showing growing multidrug resistance against most commonly used antibiotics in India [17–19]. Some latest reports emerging from different parts of India have shown an alarming rise of aminoglycoside drug resistance. One of such studies has shown high level resistance to gentamicin against five common aminoglycoside resistance genes* aac(6*′*)-Ie-aph(2*′′*)-Ia*,* aph(2*′′*)-Ib*,* aph(2*′′*)-Ic*,* aph(2*′′*)-Id*, and* aph(3*′*)-IIIa* [20]. Another report shows high level aminoglycoside resistance for gentamicin, streptomycin, and both antibiotics with the detection of various aminoglycoside modifying enzyme encoding genes from southern part of India [11]. The reports from central India and north India have also shown a similar trend of rising aminoglycoside resistant isolates [21–24]. Studies from different parts of India show increasing resistance to aminoglycoside drugs among the bacterial isolates and more and more genes are being identified and found responsible for molecular mechanism of the drug resistance. This study now confirmed that one or more antibiotic resistance genes are carried on an R plasmid and more than one antibiotic could be a substrate for certain aminoglycoside modifying enzymes. The presence of different aminoglycoside resistance genes, namely,* rrs*,* aacC2*,* aacA-aphD*, and* aphA3*, in* E. coli* infers towards emergence of defence mechanism at molecular levels. Amikacin was observed to be the most potent drug among the used antibiotics. The results clearly infer towards the expression of aminoglycoside resistance genes in the* E. coli* isolates under study from India, which is a real problem and a challenge for urinary tract infection patients. The absence of genes in the five isolates can plausibly be explained by the presence of genes for which we did not include primers or the genes that are as yet unknown. Such a resistance would limit the choices of antibiotics available to clinicians to treat bacterial infections in patients. Earlier studies in India have shown the presence of* aac(6)*,* ant(2)*, and* aph(3)* genes in the* E. coli*,* Klebsiella* species, and* Pseudomonas *species bacterial isolates [23]. A latest study has shown an array of AMEs expressed by* aac(6*′*)-Ie-aph(2*′′*)-Ia* and* aph(3*′*)-IIIa* genes responsible for causing high level of resistance in* Enterococcus *species against aminoglycosides [11]. It is clear that* E. coli* has assumed resistant patterns with the advancement of time and due to the exposure to the drugs. The resistance mechanisms lead to a compromising position for a clinician whether to subscribe the drugs to the patients or not. Various workers have developed combinational drugs with better bactericidal efficiencies and they may be a better choice for the treatment of infectious diseases caused by* E. coli*. This may be the only way to discourage the proliferation of antibiotic resistant microbes and to safeguard effectiveness of existing antimicrobial drugs. The concern of aminoglycoside resistance is serious and needs urgent attention. Regular surveillance of antimicrobial susceptibilities and effective management of bacterial infections needs urgent attention to limit the further spread of multidrug resistance in India.

## Figures and Tables

**Table 1:** Primer specification.

Name	Subfamily	Primer sequence (5→3)	PCR product
*rrs*	*rrs1*	GGATTAGATACCCTGGTAGTCC	320
	*rrs2*	TCGTTGCGGGACTTAACCCAAC	

*aacC*	*aacC1-1*	ACCTACTCCCAACATCAGCC	169
	*aacC1-2*	ATATAGATCTCACTACGCGC	
	*aacC2-1*	ACTGTGATGGGATACGCGTC	237
	*aacC2-2*	CTCCGTCAGCGTTTCAGCTA	
	*aacC3-1*	CACAAGAACGTGGTCCGCTA	185
	*aacC3-2*	AACAGGTAAGCATCCGCATC	
	*aacC4-1*	CTTCAGGATGGCAAGTTGGT	286
	*aacC4-2*	TCATCTCGTTCTCCGCTCAT	
	*aadC-1*	GCAAGGACCGACAACATTTC	165
	*aadC-2*	TGGCACAGATGGTCATAACC	

*aacA-aphD*	*aacA-aphD-1 *	CCAAGAGCAATAAGGGCATA	220
	*aacA-aphD-2*	CACTATCATAACCACTACCC	

*aphA3*	*aphA3-1*	GCCGATGTGGATTGCGAAAA	292
	*aphA3-2*	GCTTGATCCCCAGTAAGTC	
